# What Happened? Alcohol, Memory Blackouts, and the Brain

**Published:** 2003

**Authors:** Aaron M. White

**Affiliations:** Aaron M. White, Ph.D., is an assistant research professor in the Department of Psychiatry, Duke University Medical Center, Durham, North Carolina

**Keywords:** alcoholic blackout, memory interference, AOD (alcohol and other drug) intoxication, AODE (alcohol and other drug effects), AODR (alcohol and other drug related) mental disorder, long-term memory, short-term memory, state-dependent memory, BAC level, social AOD use, drug interaction, disease susceptibility, hippocampus, frontal cortex, neuroimaging, long-term potentiation

## Abstract

Alcohol primarily interferes with the ability to form new long-term memories, leaving intact previously established long-term memories and the ability to keep new information active in memory for brief periods. As the amount of alcohol consumed increases, so does the magnitude of the memory impairments. Large amounts of alcohol, particularly if consumed rapidly, can produce partial (i.e., fragmentary) or complete (i.e., en bloc) blackouts, which are periods of memory loss for events that transpired while a person was drinking. Blackouts are much more common among social drinkers—including college drinkers—than was previously assumed, and have been found to encompass events ranging from conversations to intercourse. Mechanisms underlying alcohol-induced memory impairments include disruption of activity in the hippocampus, a brain region that plays a central role in the formation of new auotbiographical memories.

If recreational drugs were tools, alcohol would be a sledgehammer. Few cognitive functions or behaviors escape the impact of alcohol, a fact that has long been recognized in the literature. As Fleming stated nearly 70 years ago, “the striking and inescapable impression one gets from a review of acute alcoholic intoxication is of the almost infinite diversity of symptoms that may ensue from the action of this single toxic agent” (1935) (pp. 94–95). In addition to impairing balance, motor coordination, decisionmaking, and a litany of other functions, alcohol produces detectable memory impairments beginning after just one or two drinks. As the dose increases, so does the magnitude of the memory impairments. Under certain circumstances, alcohol can disrupt or completely block the ability to form memories for events that transpire while a person is intoxicated, a type of impairment known as a blackout. This article reviews what is currently known regarding the specific features of acute alcohol-induced memory dysfunction, particularly alcohol-induced blackouts, and the pharmacological mechanisms underlying them.

## Effects of Alcohol on Memory

To evaluate the effects of alcohol, or any other drug, on memory, one must first identify a model of memory formation and storage to use as a reference. One classic, often-cited model, initially proposed by Atkinson and Shiffrin (1968), posits that memory formation and storage take place in several stages, proceeding from sensory memory (which lasts up to a few seconds) to short-term memory (which lasts from seconds to minutes depending upon whether the information is rehearsed) to long-term storage. This model often is referred to as the *modal model of memory,* as it captures key elements of several other major models. Indeed, elements of this model still can be seen in virtually all models of memory formation.

In the modal model of memory, when one attends to sensory information, it is transferred from a sensory memory store to short-term memory. The likelihood that information will be transferred from short-term to long-term storage, or be *encoded* into long-term memory, was once thought to depend primarily on how long the person keeps the information active in short-term memory via rehearsal. Although rehearsal clearly influences the transfer of information into long-term storage, it is important to note that other factors, such as the depth of processing (i.e., the level of true understanding and manipulation of the information), attention, motivation, and arousal also play important roles (Craik and Lockhart 1972; Otten et al. 2001; Eichenbaum 2002).^1^

**Figure 1:**
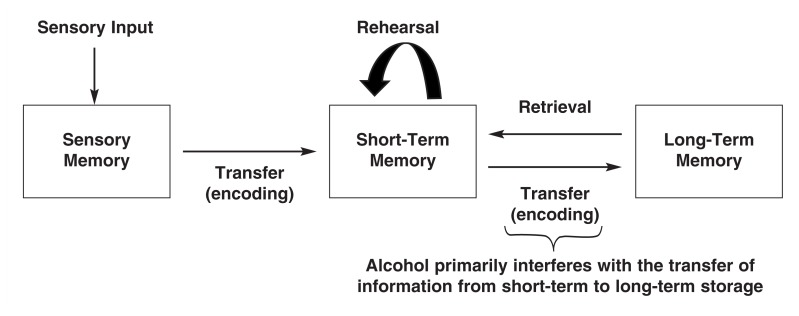
A general model of memory formation, storage, and retrieval based on the *modal model of memory* originally proposed by Atkinson and Shiffrin (1968). Alcohol seems to influence most stages of the process to some degree, but its primary effect appears to be on the transfer of information from short-term to long-term storage. Intoxicated subjects are typically able to recall information immediately after it is presented and even keep it active in short-term memory for 1 minute or more if they are not distracted. Subjects also are normally able to recall long-term memories formed before they became intoxicated; however, beginning with just one or two drinks, subjects begin to show impairments in the ability to transfer information into long-term storage. Under some circumstances, alcohol can impact this process so severely that, once sober again, subjects are unable to recall critical elements of events, or even entire events, that occurred while they were intoxicated. These impairments are known as blackouts.

Variability in the use of terms, particularly in operational definitions of short-term memory, makes it difficult to formulate a simple synopsis of the literature on alcohol-induced memory impairments. As Mello (1973) stated three decades ago with regard to the memory literature in general, “The inconsistent use of descriptive terms has been a recurrent source of confusion in the ‘short-term’ memory literature and ‘short-term’ memory has been variously defined as 5 seconds, 5 minutes, and 30 minutes” (p. 333). In spite of this inconsistency, several conclusions can be drawn from research on alcohol-induced memory impairments. One conclusion is that the impact of alcohol on the formation of new long-term “explicit” memories—that is, memories of facts (e.g., names and phone numbers) and events—is far greater than the drug’s impact on the ability to recall previously established memories or to hold new information in short-term memory (Lister et al. 1991). (See figure 1 for a diagram depicting the stages of memory and where alcohol interferes with memory.) Intoxicated subjects are typically able to repeat new information immediately after its presentation and often can keep it active in short-term storage for up to a few minutes if they are not distracted (for an early review, see Ryback 1971), though this is not always the case (Nordby et al. 1999). Similarly, subjects normally are capable of retrieving information placed in long-term storage prior to acute intoxication. In contrast, alcohol impairs the ability to store information across delays longer than a few seconds if subjects are distracted between the time they are given the new information and the time they are tested. In a classic study, Parker and colleagues (1976) reported that when intoxicated subjects were presented with “paired associates”—for example, the letter “B” paired with the month “January”—they were impaired when asked to recall the items after delays of a minute or more. However, subjects could recall paired associates that they had learned before becoming intoxicated. More recently, Acheson and colleagues (1998) observed that intoxicated subjects could recall items on word lists immediately after the lists were presented but were impaired when asked to recall the items 20 minutes later.

Ryback (1971) characterized the impact of alcohol on memory formation as a dose-related continuum, with minor impairments at one end and large impairments at the other, all impairments representing the same fundamental deficit in the ability to transfer new information from short-term to long-term storage. When doses of alcohol are small to moderate (producing blood alcohol concentrations [BACs] below 0.15 percent), memory impairments tend to be small to moderate as well. At these levels, alcohol produces what Ryback (1971) referred to as cocktail party memory deficits, lapses in memory that people might experience after having a few drinks at a cocktail party, often manifested as problems remembering what another person said or where they were in conversation. Several studies have revealed that alcohol at such levels causes difficulty forming memories for items on word lists or learning to recognize new faces (Westrick et al. 1988; Mintzer and Griffiths 2002). As the dose increases, the resulting memory impairments can become much more profound, sometimes culminating in blackouts—periods for which a person is unable to remember critical elements of events, or even entire events, that occurred while he or she was intoxicated.

### Alcohol-Induced Blackouts

Blackouts represent episodes of amnesia, during which subjects are capable of participating even in salient, emotionally charged events—as well as more mundane events—that they later cannot remember (Goodwin 1995). Like milder alcohol-induced memory impairments, these periods of amnesia are primarily “anterograde,” meaning that alcohol impairs the ability to form new memories while the person is intoxicated, but does not typically erase memories formed before intoxication. Formal research into the nature of alcohol-induced blackouts began in the 1940s with the work of E.M. Jellinek (1946). Jellinek’s initial characterization of blackouts was based on data collected from a survey of Alcoholics Anonymous members. Noting that recovering alcoholics frequently reported having experienced alcohol-induced amnesia while they were drinking, Jellinek concluded that the occurrence of blackouts is a powerful indicator of alcoholism.

In 1969, Goodwin and colleagues published two of the most influential studies in the literature on blackouts (Goodwin et al. 1969*a*,*b*). Based on interviews with 100 hospitalized alcoholics, 64 of whom had a history of blackouts, the authors posited the existence of two qualitatively different types of blackouts: en bloc and fragmentary blackouts. People experiencing en bloc blackouts are unable to recall any details whatsoever from events that occurred while they were intoxicated, despite all efforts by the drinkers or others to cue recall. Referring back to our general model of memory formation, it is as if the process of transferring information from short-term to long-term storage has been completely blocked. En bloc memory impairments tend to have a distinct onset. It is usually less clear when these blackouts end because people typically fall asleep before they are over. Interestingly, people appear able to keep information active in short-term memory for at least a few seconds. As a result, they can often carry on conversations, drive automobiles, and engage in other complicated behaviors. Information pertaining to these events is simply not transferred into long-term storage. Ryback (1970) wrote that intoxicated subjects in one of his studies “could carry on conversations during the amnesic state, but could not remember what they said or did 5 minutes earlier. Their immediate and remote memory were intact” (p. 1003). Similarly, in their study of memory impairments in intoxicated alcoholics, Goodwin and colleagues (1970) reported that subjects who experienced blackouts for testing sessions showed intact memory for up to 2 minutes while the sessions were taking place.

Unlike en bloc blackouts, fragmentary blackouts involve partial blocking of memory formation for events that occurred while the person was intoxicated. Goodwin and colleagues (1969*a*) reported that subjects experiencing fragmentary blackouts often become aware that they are missing pieces of events only after being reminded that the events occurred. Interestingly, these reminders trigger at least some recall of the initially missing information. Research suggests that fragmentary blackouts are far more common than those of the en bloc variety (White et al. 2004; Hartzler and Fromme 2003*b*; Goodwin et al. 1969*b*).

### Blackouts: State-Dependent Memory Formation?

Early anecdotal evidence suggested that blackouts might actually reflect state-dependent information storage—that is, people might be able to remember events that occurred while they were intoxicated if they returned to that state (e.g., Goodwin et al. 1969*a*). State-dependent memory can be viewed as a special case of a broader category known as context-dependent memory (e.g., White et al. 2002*a*), in which cues that are associated with an event when a memory is formed tend to help trigger recall for that event at a later time. For instance, in a classic study by Godden and Baddeley (1975) divers who learned word lists either on land or under water remembered more words when tested in the same context in which learning took place (i.e., land–land or water–water). Likewise, returning to the same emotional or physiological state that was present when a memory was formed often can facilitate recall of that memory. It is not uncommon to hear stories of drinkers who stash alcohol or money while intoxicated and can locate the hiding places only after becoming intoxicated again (Goodwin 1995). Regardless of how compelling such stories can be, clear evidence of state-dependent learning under the influence of alcohol is lacking. In one recent study, Weissenborn and Duka (2000) examined whether subjects who learned word lists while intoxicated could recall more items if they were intoxicated again during the testing session. No such state-dependency was observed. Similarly, Lisman (1974) tried unsuccessfully to help subjects resurrect lost information for events occurring during periods of intoxication by getting them intoxicated once again.

### Blood Alcohol Concentrations and Blackouts

Drinking large quantities of alcohol often precedes blackouts, but several other factors also appear to play important roles in causing such episodes of memory loss. As Goodwin and colleagues (1969*a*) stated with regard to subjects in one of their studies, “Although blackouts almost always were associated with heavy drinking, this alone seemed insufficient to produce one. On many other occasions, subjects said they had drunk as much or more without memory loss” (p. 195). Among the factors that preceded blackouts were gulping drinks and drinking on an empty stomach, each of which leads to a rapid rise in BAC.

Subsequent research provided additional evidence suggesting a link between blackouts and rapidly rising BACs. Goodwin and colleagues (1970) examined the impact of acute alcohol exposure on memory formation in a laboratory setting. The author recruited 10 male subjects for the project, all but one through the unemployment office in St. Louis, Missouri. Most subjects met diagnostic criteria for alcoholism and half had a history of frequent blackouts. The men were asked to consume roughly 16 to 18 ounces of 86-proof bourbon in approximately 4 hours. Beginning 1 hour after subjects began drinking, memory was tested by presenting subjects with several different stimuli, including a series of children’s toys and scenes from erotic films. Subjects were asked to recall details regarding these stimuli 2 minutes, 30 minutes, and 24 hours after the stimuli were shown. Half of the subjects reported no recall for the stimuli or their presentation 30 minutes and 24 hours after the events, though most seemed to recall the stimuli 2 minutes after presentation. Lack of recall for the events 24 hours later, while sober, represents clear experimental evidence for the occurrence of blackouts. The fact that subjects could remember aspects of the events 2 minutes after they occurred but not 30 minutes or 24 hours afterward provides compelling evidence that the blackouts stemmed from an inability to transfer information from short-term to long-term storage. For all but one subject in the blackout group, memory impairments began during the first few hours of drinking, when BAC levels were still rising. The average peak BAC in this group, which was roughly 0.28 percent, occurred approximately 2.5 hours after the onset of drinking.

In a similar study, Ryback (1970) examined the impact of alcohol on memory in seven hospitalized alcoholics given access to alcohol over the course of several days. All subjects were White males between the ages of 31 and 44. Blackouts occurred in five of the seven subjects, as evidenced by an inability to recall salient events that occurred while drinking the day before (e.g., one subject could not recall preparing to hit another over the head with a chair). Estimates of BAC levels during blackout periods suggested that they often began at levels around 0.20 percent and as low as 0.14 percent. The duration of blackouts ranged from 9 hours to 3 days. Based on his observations, Ryback concluded that a key predictor of blackouts was the rate at which subjects consumed their drinks. He stated, “It is important to note that all the blackout periods occurred after a rapid rise in blood alcohol level” (p. 622). The two subjects who did not black out, despite becoming extremely intoxicated, experienced slow increases in blood alcohol levels.

### Blackouts Among Social Drinkers

Most of the research conducted on blackouts during the past 50 years has involved surveys, interviews, and direct observation of middle-aged, primarily male alcoholics, many of whom were hospitalized. Researchers have largely ignored the occurrence of blackouts among young social drinkers, so the idea that blackouts are an unlikely consequence of heavy drinking in nonalcoholics has remained deeply entrenched in both the scientific and popular cultures. Yet there is clear evidence that blackouts do occur among social drinkers. Knight and colleagues (1999) observed that 35 percent of trainees in a large pediatric residency program had experienced at least one blackout. Similarly, Goodwin (1995) reported that 33 percent of the first-year medical students he interviewed acknowledged having had at least one blackout. “They were inexperienced,” he wrote. “They drank too much too quickly, their blood levels rose extremely quickly, and they experienced amnesia” (p. 315). In a study of 2,076 Finnish males, Poikolainen (1982) found that 35 percent of all males surveyed had had at least one blackout in the year before the survey.

As might be expected given the excessive drinking habits of many college students (Wechsler et al. 2002), this population commonly experiences blackouts. White and colleagues (2002*c*) recently surveyed 772 undergraduates regarding their experiences with blackouts. Respondents who answered yes to the question “Have you ever awoken after a night of drinking not able to remember things that you did or places that you went?” were considered to have experienced blackouts. Fifty-one percent of the students who had ever consumed alcohol reported blacking out at some point in their lives, and 40 percent reported experiencing a blackout in the year before the survey. Of those who had consumed alcohol during the 2 weeks before the survey, 9.4 percent reported blacking out during this period. Students in the study reported that they later learned that they had participated in a wide range of events they did not remember, including such significant activities as vandalism, unprotected intercourse, driving an automobile, and spending money.

During the 2 weeks preceding the survey, an equal percentage of males and females experienced blackouts, despite the fact that males drank significantly more often and more heavily than females. This outcome suggests that at any given level of alcohol consumption, females—a group infrequently studied in the literature on blackouts—are at greater risk than males for experiencing blackouts. The greater tendency of females to black out likely arises, in part, from well-known gender differences in physiological factors that affect alcohol distribution and metabolism, such as body weight, proportion of body fat, and levels of key enzymes. There also is some evidence that females are more susceptible than males to milder forms of alcohol-induced memory impairments, even when given comparable doses of alcohol (Mumenthaler et al. 1999).

In a subsequent study, White and colleagues (2004) interviewed 50 undergraduate students, all of whom had experienced at least one blackout, to gather more information about the factors related to blackouts. As in the previous study, students reported engaging in a range of risky behaviors during blackouts, including sexual activity with both acquaintances and strangers, vandalism, getting into arguments and fights, and others. During the night of their most recent blackout, most students drank either liquor alone or in combination with beer. Only 1 student out of 50 reported that the most recent blackout occurred after drinking beer alone. On average, students estimated that they consumed roughly 11.5 drinks before the onset of the blackout. Males reported drinking significantly more than females, but they did so over a significantly longer period of time. As a result, estimated peak BACs during the night of the last blackout were similar for males (0.30 percent) and females (0.35 percent). As Goodwin observed in his work with alcoholics (1969b), fragmentary blackouts occurred far more often than en bloc blackouts, with four out of five students indicating that they eventually recalled bits and pieces of the events. Roughly half of all students (52 percent) indicated that their first full memory after the onset of the blackout was of waking up in the morning, often in an unfamiliar location. Many students, more females (59 percent) than males (25 percent), were frightened by their last blackout and changed their drinking habits as a result.

### Use of Other Drugs During Blackouts

Alcohol interacts with several other drugs, many of which are capable of producing amnesia on their own. For instance, diazepam (Valium^®^) and flunitrazepam (Rohypnol) are benzodiazepine sedatives that can produce severe memory impairments at high doses (White et al. 1997; Saum and Inciardia 1997). Alcohol enhances the effects of benzodiazepines (for a review, see Silvers et al. 2003). Thus, combining these compounds with alcohol could dramatically increase the likelihood of experiencing memory impairments. Similarly, the combination of alcohol and THC, the primary psychoactive compound in marijuana, produces greater memory impairments than when either drug is given alone (Ciccocioppo et al. 2002). Given that many college students use other drugs in combination with alcohol (O’Malley and Johnston 2002), some of the blackouts reported by students may arise from polysubstance use rather than from alcohol alone. Indeed, based on interviews with 136 heavy-drinking young adults (mean age 22), Hartzler and Fromme (2003*b*) concluded that en bloc blackouts often arise from the combined use of alcohol and other drugs. White and colleagues (2004) observed that, among 50 undergraduate students with a history of blackouts, only 3 students reported using other drugs during the night of their most recent blackout, and marijuana was the drug in each case.

### Are Some People More Likely Than Others to Experience Blackouts?

In classic studies of hospitalized alcoholics by Goodwin and colleagues (1969a,b), 36 out of the 100 patients interviewed indicated that they had never experienced a blackout. In some ways, the patients who did not experience blackouts are as interesting as the patients who did. What was it about these 36 patients that kept them from blacking out, despite the fact that their alcoholism was so severe that it required hospitalization? Although they may actually have experienced blackouts but simply were unaware of them, there may have been something fundamentally different about these patients that diminished their likelihood of experiencing memory impairments while drinking.

In support of this possibility, a recent study by Hartzler and Fromme (2003*a*) suggests that people with a history of blackouts are more vulnerable to the effects of alcohol on memory than those without a history of blackouts. These authors recruited 108 college students, half of whom had experienced at least one fragmentary blackout in the previous year. While sober, members of the two groups performed comparably in memory tasks. However, when they were mildly intoxicated (0.08 percent BAC) those with a history of fragmentary blackouts performed worse than those without such a history. There are two possible interpretations for these data, both of which support the hypothesis that some people are more susceptible to blackouts than others. One plausible interpretation is that subjects in the fragmentary blackout group always have been more vulnerable to alcohol-induced memory impairments, which is why they performed poorly during testing under alcohol, and why they are members of the blackout group in the first place. A second interpretation is that subjects in the blackout group performed poorly during testing as a result of drinking enough in the past to experience alcohol-induced memory impairments. In other words, perhaps their prior exposure to alcohol damaged the brain in a way that predisposed them to experiencing future memory impairments. This latter possibility is made more likely by recent evidence that students who engage in repeated episodes of heavy, or binge, drinking are more likely than other students to exhibit memory impairments when they are intoxicated (Weissenborn and Duka 2000). Similar results have been observed in animal studies (White et al. 2000*a*).

The argument for an inherent vulnerability to alcohol-induced memory impairments, including blackouts, is strengthened by two recent studies. In an impressive longitudinal study, Baer and colleagues (2003) examined the drinking habits of pregnant women in 1974 and 1975, and then studied alcohol use and related problems in their offspring at seven different time points during the following 21 years. These authors observed that prenatal alcohol exposure was associated with increased rates of experiencing alcohol-related consequences, including blackouts, even after controlling for the offsprings’ general drinking habits. In addition, a recent report by Nelson and colleagues (2004) suggests that there might actually be a genetic contribution to the susceptibility to blackouts, indicating that some people simply are built in a way that makes them more vulnerable to alcohol-induced amnesia.

As discussed in the section below on the potential brain mechanisms underlying alcohol-induced amnesia, it is easy to imagine that the impact of alcohol on brain circuitry could vary from person to person, rendering some people more sensitive than others to the memory-impairing effects of the drug.

## How Does Alcohol Impair Memory?

During the first half of the 20th century, two theoretical hurdles hampered progress toward an understanding of the mechanisms underlying the effects of alcohol on memory. More recent research has cleared away these hurdles, allowing for tremendous gains in the area during the past 50 years.

The first hurdle concerned scientists’ understanding of the functional neuroanatomy of memory. In the 1950s, following observations of an amnesic patient known as H.M., it became clear that different brain regions are involved in the formation, storage, and retrieval of different types of memory. In 1953, large portions of H.M.’s medial temporal lobes, including most of his hippocampus, were removed in an effort to control intractable seizures (Scoville and Milner 1957). Although the frequency and severity of H.M.’s seizures were significantly reduced by the surgery, it soon became clear that H.M. suffered from a dramatic syndrome of memory impairments. He still was able to learn basic motor skills, keep information active in short-term memory for a few seconds or more if left undistracted, and remember episodes of his life from long ago, but he was unable to form new long-term memories for facts and events. The pattern of H.M.’s impairments also forced a re-examination of models of long-term memory storage. Specifically, although H.M. was able to retrieve long-term memories formed roughly a year or more before his surgery, he could not recall events that transpired within the year preceding his surgery. This strongly suggests that the transfer of information into long-term storage actually takes place over several years, with the hippocampus being necessary for its retrieval for the first year or so.

**Figure 2:**
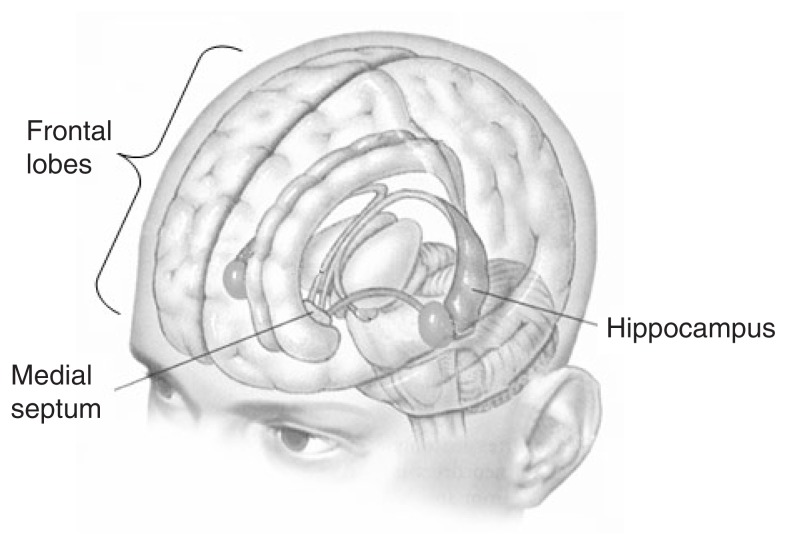
The human brain, showing the location of the hippocampus, the frontal lobes, and the medial septum.

Subsequent research with other patients confirmed that the hippocampus, an irregularly shaped structure deep in the forebrain, is critically involved in the formation of memories for events (see figure 2 for a depiction of the brain, with the hippocampus and other relevant structures highlighted). Patient R.B. lost a significant amount of blood as a result of heart surgery. He survived but showed memory impairments similar to those exhibited by H.M. Upon his death, histology revealed that the loss of blood to R.B.’s brain damaged a small region of the hippocampus called hippocampal area CA1, which contains neurons known as pyramidal cells because of the triangular shape of their cell bodies (Zola-Morgan et al. 1986). Hippocampal CA1 pyramidal cells assist the hippocampus in communicating with other areas of the brain. The hippocampus receives information from a wide variety of brain regions, many of them located in the tissue, called the neocortex, that blankets the brain and surrounds other brain structures. (Neocortex literally means “new bark” or “new covering.” When one looks at a picture of the human brain, most of what is visible is neocortex.) The hippocampus somehow ties information from other brain regions together to form new autobiographical memories, and CA1 pyramidal cells send the results of this processing back out to the neocortex. As is clear from patient R.B., removing CA1 pyramidal cells from the circuitry prevents the hippocampal memory system from doing its job.

The second barrier to understanding the mechanisms underlying alcohol’s effects on memory was an incomplete understanding of how alcohol affects brain function at a cellular level. Until recently, alcohol was assumed to affect the brain in a general way, simply shutting down the activity of all cells with which it came in contact. The pervasiveness of this assumption is reflected in numerous writings during the early 20th century. For instance, Fleming (1935) wrote, “The prophetic generalization of Schmiedeberg in 1833 that the pharmacological action of alcohol on the cerebrum is purely depressant has been found, most pharmacologists will agree, to characterize its action in general on all tissues” (p. 89). During the 1970s, researchers hypothesized that alcohol depressed neural activity by altering the movement of key molecules (in particular, lipids) in nerve cell membranes. This change then led to alterations in the activity of proteins, including those that influence communication between neurons by controlling the passage of positively or negatively charged atoms (i.e., ions) through cell membranes (e.g., Chin and Goldstein 1977). This view persisted into the late 1980s, at which time the consensus began to shift as evidence mounted that alcohol has selective effects on the brain’s nerve-cell communication (i.e., neurotransmitter) systems, altering activity in some types of receptors but not others (e.g., Criswell et al. 1993). Substantial evidence now indicates that alcohol selectively alters the activity of specific complexes of proteins embedded in the membranes of cells (i.e., receptors) that bind neurotransmitters such as gamma-aminobutyric acid (GABA), glutamate, serotonin, acetylcholine, and glycine (for a review, see Little 1999). In some cases, only a few amino acids appear to distinguish receptors that are sensitive to alcohol from those that are not (Peoples and Stewart 2000). It remains unclear exactly how alcohol interacts with receptors to alter their activity.

### Alcohol, Memory, and the Hippocampus

More than 30 years ago, both Ryback (1970) and Goodwin and colleagues (1969*a*) speculated that alcohol might impair memory formation by disrupting activity in the hippocampus. This speculation was based on the observation that acute alcohol exposure (in humans) produces a syndrome of memory impairments similar in many ways to the impairments produced by hippocampal damage. Specifically, both acute alcohol exposure and hippocampal damage impair the ability to form new long-term, explicit memories but do not affect short-term memory storage or, in general, the recall of information from long-term storage.

Research conducted in the past few decades using animal models supports the hypothesis that alcohol impairs memory formation, at least in part, by disrupting activity in the hippocampus (for a review, see White et al. 2000*b*). Such research has included behavioral observation; examination of slices of and brain tissue, neurons in cell culture, and brain activity in anesthetized or freely behaving animals; and a variety of pharmacological techniques.

As mentioned above, damage limited to the CA1 region of the hippocampus dramatically disrupts the ability to form new explicit memories (Zola-Morgan et al. 1986). In rodents, the actions of CA1 pyramidal cells have striking behavioral correlates. Some cells tend to discharge electrical signals that result in one cell communicating with other cells (i.e., action potentials) when the rodent is in a distinct location in its environment. The location differs for each cell. For instance, while a rat searches for food on a plus-shaped maze, one pyramidal cell might generate action potentials primarily when the rat is at the far end of the north arm, while another might generate action potentials primarily when the rat is in the middle of the south arm, and so on. Collectively, the cells that are active in that particular environment create a spatial, or contextual map that serves as a framework for event memories created in that environment. Because of the location-specific firing of these cells, they often are referred to as “place-cells,” and the regions of the environment in which they fire are referred to as “place-fields” (for reviews, see Best and White 1998; Best et al. 2001). Given that CA1 pyramidal cells are critically important to the formation of memories for facts and events, and the clear behavioral correlates of their activity in rodents, it is possible to assess the impact of alcohol on hippocampal output in an intact, fully functional brain by studying these cells.

**Figure 3:**
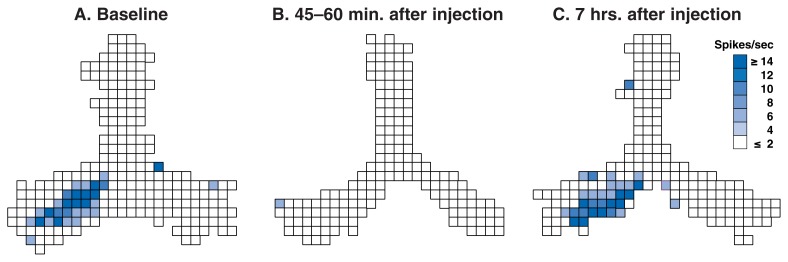
Alcohol suppresses hippocampal pyramidal cell activity in an awake, freely behaving rat. Pyramidal cells often fire when the animal is in discrete regions of its environment, earning them the title “place-cells.” The specific areas of the environment where these cells fire are referred to as place-fields. The figure shows the activity of an individual pyramidal cell before alcohol administration (baseline), 45 to 60 minutes after alcohol administration, and 7 hours after alcohol administration (1.5 g/kg). Each frame in the figure shows the firing rate and firing location of the cell across a 15-minute block of time during which the rat was foraging for food on a symmetric, Y-shaped maze. White pixels are pixels in which the cell fired at very low rates, and darker colors represent higher firing rates (see key to the right of figure). As is clear from a comparison of activity during baseline and 45 to 60 minutes after alcohol administration, the activity of the cell was essentially shut off by alcohol. Neural activity returned to near normal levels within roughly 7 hours after alcohol administration.

In recent work with awake, freely behaving rats, White and Best (2000) showed that alcohol profoundly suppresses the activity of pyramidal cells in region CA1. The researchers allowed the rats to forage for food for 15 minutes in a symmetric, Y-shaped maze and measured the animals’ hippocampal activity using tiny wires (i.e., microelectrodes) implanted in their brains. Figure 3 displays the activity of an individual CA1 pyramidal cell. The activity—which corresponds to the middle portion of the lower left arm of the maze—is shown before alcohol administration (A), 45 to 60 minutes after alcohol administration (B), and 7 hours after alcohol administration (C). The dose of alcohol used in the testing session was 1.5 grams per kilogram of body weight— enough to produce a peak BAC of about 0.16 percent. (A corresponding BAC in humans would be twice the legal driving limit in most States.) As the figure illustrates, the cell’s activity was essentially shut off by alcohol. Neural activity returned to near-normal levels within about 7 hours of alcohol administration.

White and Best administered several doses of alcohol in this study, ranging from 0.5 g/kg to 1.5 g/kg. (Only one of the experiments is represented in figure 3.) They found that the dose affected the degree of pyramidal cell suppression. Although 0.5 g/kg did not produce a significant change in the firing of hippocampal pyramidal cells, 1.0 and 1.5 g/kg produced significant suppression of firing during a 1-hour testing session following alcohol administration. The dose-dependent suppression of CA1 pyramidal cells is consistent with the dose-dependent effects of alcohol on episodic memory formation.

### Alcohol and Hippocampal Long-Term Potentiation

In addition to suppressing the output from pyramidal cells, alcohol has several other effects on hippocampal function. For instance, alcohol severely disrupts the ability of neurons to establish long-lasting, heightened responsiveness to signals from other cells (Bliss and Collinridge 1993). This heightened responsiveness is known as long-term potentiation (LTP). Because researchers have theorized that something like LTP occurs naturally in the brain during learning (for a review, see Martin and Morris 2002), many investigators have used LTP as a model for studying the neurobiology underlying the effects of drugs, including alcohol, on memory.

In a typical LTP experiment, two electrodes (A and B) are lowered into a slice of hippocampal tissue kept alive by bathing it in oxygenated artificial cerebral spinal fluid (ACSF). A small amount of current is passed through electrode A, causing the neurons in this area to send signals to cells located near electrode B. Electrode B then is used to record how the cells in the area respond to the incoming signals. This response is the baseline response. Next, a specific pattern of stimulation intended to model the pattern of activity that might occur during an actual learning event is delivered through electrode A. When the original stimulus that elicited the baseline response is delivered again through electrode A, the response recorded at electrode B is larger (i.e., potentiated). In other words, as a result of the patterned input, cells at position B now are more responsive to signals sent from cells at position A. The potentiated response often lasts for an extended period of time, hence the term *long-term potentiation*.

Alcohol interferes with the establishment of LTP (Morrisett and Swartzwelder 1993; Givens and McMahon 1995; Pyapali et al. 1999; Schummers and Browning 2001), and this impairment begins at concentrations equivalent to those produced by consuming just one or two standard drinks (e.g., a 12-oz beer, 1.5-oz of liquor in a shot or mixed drink, or a 5-oz glass of wine) (Blitzer et al. 1990). If sufficient alcohol is present in the ACSF bathing the slice of hippocampal tissue when the patterned stimulation is given, the response recorded later at position B will not be larger than it was at baseline (that is, it will not be potentiated). And, just as alcohol tends not to impair recall of memories established before alcohol exposure, alcohol does not disrupt the expression of LTP established before alcohol exposure.

One of the key requirements for the establishment of LTP in the hippocampus is that a type of signal receptor known as the NMDA^2^ receptor becomes activated. Activation of the NMDA receptor allows calcium to enter the cell, which sets off a chain of events leading to long-lasting changes in the cell’s structure or function, or both. Alcohol interferes with the activation of the NMDA receptor, thereby preventing the influx of calcium and the changes that follow (Swartzwelder et al. 1995). This is believed to be the primary mechanism underlying the effects of alcohol on LTP, though other transmitter systems probably are also involved (Schummers and Browning 2001).

### Indirect Effects of Alcohol on Hippocampal Function

Like other brain regions, the hippocampus does not operate in isolation. Information processing in the hippocampus depends on coordinated input from a variety of other structures, which gives alcohol and other drugs additional opportunities to disrupt hippocampal functioning. One brain region that is central to hippocampal functioning is a small structure in the fore brain known as the medial septum (Givens et al. 2000). The medial septum sends rhythmic excitatory and inhibitory signals to the hippocampus, causing rhythmic changes in the activity of hippocampal pyramidal cells. In electroencephalograph recordings, this rhythmic activity, referred to as the theta rhythm, occurs within a frequency of roughly 6 to 9 cycles per second (hertz) in actively behaving rats. The theta rhythm is thought to act as a gatekeeper, increasing or decreasing the likelihood that information entering the hippocampus from cortical structures will be processed (Orr et al. 2001). (For more information on the role of electrophysiology in diagnosing alcohol problems, see the article in this issue by Porjesz and Begleiter.) Information entering the hippocampus when pyramidal cells are slightly excited (i.e., slightly depolarized) has a better chance of influencing hippocampal circuitry than signals that arrive when the cells are slightly suppressed (i.e., slightly hyperpolarized).

Manipulations that disrupt the theta rhythm also disrupt the ability to perform tasks that depend on the hippocampus (Givens et al. 2000). Alcohol disrupts the theta rhythm in large part by suppressing the output of signals from medial septal neurons to the hippocampus (Steffensen et al. 1993; Givens et al. 2000). Given the powerful influence that the medial septum has on information processing in the hippocampus, the impact of alcohol on cellular activity in the medial septum is likely to play an important role in the effects of alcohol on memory. Indeed, in rats, putting alcohol directly into the medial septum alone produces memory impairments (Givens and McMahon 1997).

### Other Brain Regions Involved in Alcohol-Induced Memory Impairments

The hippocampus is not the only structure involved in memory formation. A host of other brain structures also are involved in memory formation, storage, and retrieval (Eichenbaum 2002). Recent research with humans has yielded compelling evidence that key areas of the frontal lobes play important roles in short-term memory and the formation and retrieval of long-term explicit memories (e.g., Shastri 2002; Curtis and D’Esposito 2003; Ranganath et al. 2003). Damage to the frontal lobes leads to profound cognitive impairments, one of which is a difficulty forming new memories. Recent evidence suggests that memory processes in the frontal lobes and the hippocampus are coordinated via reciprocal connections (Wall and Messier 2001; Shastri 2002), raising the possibility that dysfunction in one structure could have deleterious effects on the functioning of the other.

Considerable evidence suggests that chronic alcohol use damages the frontal lobes and leads to impaired performance of tasks that rely on frontal lobe functioning (Kril and Halliday 1999; Moselhy et al. 2001). “Shrinkage” in brain volume, changes in gene expression, and disruptions in how performing certain tasks affects blood flow in the brain all have been observed in the frontal lobes of alcohol-dependent subjects (Kril and Halliday 1999; Lewohl et al. 2000; Tapert et al. 2001; Kubota et al. 2001; Desmond et al. 2003).

Although much is known about the effects of chronic (i.e., repeated) use of alcohol on frontal lobe function, little is known about the effects of one-time (i.e., acute) use of alcohol on activity in the frontal lobes, or the relationship of such effects to alcohol-induced memory impairments. Compelling evidence indicates that acute alcohol use impairs the performance of a variety of frontal lobe–mediated tasks, like those that require planning, decisionmaking, and impulse control (Weissenborn and Duka 2003; Burian et al. 2003), but the underlying mechanisms are not known. Research also suggests that baseline blood flow to the frontal lobes increases during acute intoxication (Volkow et al. 1988; Tiihonen et al. 1994), that metabolism in the frontal lobes decreases (Wang et al. 2000), and that alcohol reduces the amount of activity that occurs in the frontal lobes when the frontal lobes are exposed to pulses from a strong magnetic field (Kahkonen et al. 2003). Although the exact meaning of these changes remains unclear, the evidence suggests that acute intoxication alters the normal functioning of the frontal lobes. Future research is needed to shed more light on this important question. In particular, research in animals will be an important supplement to studies in humans, affording a better understanding of the underlying prefrontal circuitry involved in alcohol-induced memory impairment.

## Summary and Conclusions

As detailed in this brief review, alcohol can have a dramatic impact on memory. Alcohol primarily disrupts the ability to form new long-term memories; it causes less disruption of recall of previously established long-term memories or of the ability to keep new information active in short-term memory for a few seconds or more. At low doses, the impairments produced by alcohol are often subtle, though they are detectable in controlled conditions. As the amount of alcohol consumed increases, so does the magnitude of the memory impairments. Large quantities of alcohol, particularly if consumed rapidly, can produce a blackout, an interval of time for which the intoxicated person cannot recall key details of events, or even entire events. En bloc blackouts are stretches of time for which the person has no memory whatsoever. Fragmentary blackouts are episodes for which the drinker’s memory is spotty, with “islands” of memory providing some insight into what transpired, and for which more recall usually is possible if the drinker is cued by others. Blackouts are much more common among social drinkers than previously assumed and should be viewed as a potential consequence of acute intoxication regardless of age or whether one is clinically dependent upon alcohol.

Tremendous progress has been made toward an understanding of the mechanisms underlying alcohol-induced memory impairments. Alcohol disrupts activity in the hippocampus via several routes—directly, through effects on hippocampal circuitry, and indirectly, by interfering with interactions between the hippocampus and other brain regions. The impact of alcohol on the frontal lobes remains poorly understood, but probably plays an important role in alcohol-induced memory impairments.

Modern neuroimaging techniques, such as positron emission tomography (PET) and functional magnetic resonance imaging (fMRI), provide incredible opportunities for investigating the impact of drugs like alcohol on brain function during the performance of cognitive tasks. The use of these techniques will no doubt yield important information regarding the mechanisms underlying alcohol-induced memory impairments in the coming years. Memory formation and retrieval are highly influenced by factors such as attention and motivation (e.g., Kensinger et al. 2003). With the aid of neuroimaging techniques, researchers may be able to examine the impact of alcohol on brain activity related to these factors, and then determine how alcohol contributes to memory impairments.

Despite advances in human neuroimaging techniques, animal models remain absolutely essential in the study of mechanisms underlying alcohol-induced memory impairments. Hopefully, future work will reveal more regarding the ways in which the effects of alcohol on multiple transmitter systems interact to disrupt memory formation. Similarly, recent advances in electrophysiological recording techniques, which allow for recordings from hundreds of individual cells in several brain regions simultaneously (Kralik et al. 2001), could provide much-needed information regarding the impact of alcohol on the interactions between disparate brain regions involved in the encoding, storage, and retrieval of information.

## Footnotes

1 It is well beyond the scope of this review to assess the impact of alcohol on memory utilizing multiple perspectives on information processing and storage. For simplicity, this review will characterize the effects of alcohol on memory using a three-stage process of memory formation akin to the modal model. The interpretation of the effects of alcohol on memory likely would vary somewhat depending on the memory model one uses.

2 *N*-methyl-D-aspartate (NMDA) is a receptor for the neurotransmitter glutamate.

## References

[b1-186-196] Acheson S, Stein R, Swartzwelder HS (1998). Impairment of semantic and figural memory by acute ethanol: Age-dependent effects. Alcoholism: Clinical and Experimental Research.

[b2-186-196] Atkinson RC, Shiffrin RM, Spence KW (1968). Human memory: A proposed system and its control processes. The Psychology of Learning and Motivation: Advances in Research and Theory.

[b3-186-196] Baer JS, Sampson PD, Barr HM (2003). A 21-year longitudinal analysis of the effects of prenatal alcohol exposure on young adult drinking. Archives of General Psychiatry.

[b4-186-196] Best PJ, White AM (1998). Hippocampal cellular activity: A brief history of space. Proceedings of the National Academy of Sciences of the USA.

[b5-186-196] Best PJ, White AM, Minai A (2001). Spatial processing in the brain: The activity of hippocampal place-cells. Annual Review of Neuroscience.

[b6-186-196] Bliss TVP, Collingridge GL (1993). A synaptic model of memory: Long-term potentiation in the hippocampus. Nature.

[b7-186-196] Blitzer RD, Gil O, Landau EM (1990). Long-term potentiation in rat hippocampus is inhibited by low concentrations of ethanol. Brain Research.

[b8-186-196] Burian SE, Hensberry R, Liguori A (2003). Differential effects of alcohol and alcohol expectancy on risk-taking during simulated driving. Human Psychopharmacology.

[b9-186-196] Chin JH, Goldstein DB (1977). Effects of low concentrations of ethanol on the fluidity of spin-labeled erythrocyte and brain membranes. Molecular Pharmacology.

[b10-186-196] Ciccocioppo R, Antonelli L, Biondini M (2002). Memory impairment following combined exposure to delta(9)-tetrahydrocannabinol and ethanol in rats. European Journal of Pharmacology.

[b11-186-196] Craik FIM, Lockhart RS (1972). Levels of processing: A framework for memory research. J. ournal of Verbal Learning and Verbal Behavior.

[b12-186-196] Criswell HE, Simson PE, Duncan GE (1993). Molecular basis for regionally specific action of ethanol on gamma-aminobutyric acid_A_ receptors: Generalization to other ligand-gated ion channels. Journal of Pharmacology and Experimental Therapeutics.

[b13-186-196] Curtis CE, D’Esposito M (2003). Persistent activity in the prefrontal cortex during working memory. Trends in Cognitive Sciences.

[b14-186-196] Desmond JE, Chen SH, DeRosa E (2003). Increased frontocerebellar activation in alcoholics during verbal working memory: An fMRI study. NeuroImage.

[b15-186-196] Eichenbaum H (2002). The Cognitive Neuroscience of Memory: An Introduction.

[b16-186-196] Fleming R (1935). A psychiatric concept of acute alcoholic intoxication. American Journal of Psychiatry.

[b17-186-196] Givens B, McMahon K (1995). Ethanol suppresses the induction of long-term potentiation in vivo. Brain Research.

[b18-186-196] Givens B, McMahon K (1997). Effects of ethanol on nonspatial working memory and attention in rats. Behavioral Neuroscience.

[b19-186-196] Givens B, Williams JM, Gill TM (2000). Septohippocampal pathway as a site for the memory-impairing effects of ethanol. Hippocampus.

[b20-186-196] Godden DR, Baddeley AD (1975). Context-dependent memory in two natural environments: On land and under water. British Journal of Psychology.

[b21-186-196] Goodwin DW (1995). Alcohol amnesia. Addiction.

[b22-186-196] Goodwin DW Crane JB, Guze SB (1969a). Alcoholic “blackouts”: A review and clinical study of 100 alcoholics. American Journal of Psychiatry.

[b23-186-196] Goodwin DW, Crane JB, Guze SB (1969b). Phenomenological aspects of the alcoholic “blackout.”. British Journal of Psychiatry.

[b24-186-196] Goodwin DW, Othmer E, Halikas JA (1970). Loss of short-term memory as a predictor of the alcoholic “black-out.”. Nature.

[b25-186-196] Hartzler B, Fromme K (2003a). Fragmentary blackouts: Their etiology and effect on alcohol expectancies. Alcoholism: Clinical and Experimental Research.

[b26-186-196] Hartzler B, Fromme K (2003b). Fragmentary and en bloc blackouts: Similarity and distinction among episodes of alcohol-induced memory loss. Journal of Studies on Alcohol.

[b27-186-196] Jellinek EM (1946). Phases in the drinking history of alcoholics: Analysis of a survey conducted by the official organ of Alcoholics Anonymous. Quarterly Journal of Studies on Alcohol.

[b28-186-196] Kahkonen S, Wilenius J, Nikulin VV (2003). Alcohol reduces prefrontal cortical excitability in humans: A combined TMS and EEG study. Neuropsychopharmacology.

[b29-186-196] Kensinger EA, Clarke RJ, Corkin S (2003). What neural correlates underlie successful encoding and retrieval? A functional magnetic resonance imaging study using a divided attention paradigm. Journal of Neuroscience.

[b30-186-196] Knight JR, Palacios JN, Shannon M (1999). Prevalence of alcohol problems among pediatric residents. Archives of Pediatrics & Adolescent Medicine.

[b31-186-196] Kralik JD, Dimitrov DF, Krupa DJ (2001). Techniques for long-term multisite neuronal ensemble recordings in behaving animals. Methods.

[b32-186-196] Kril JJ, Halliday GM (1999). Brain shrinkage in alcoholics: A decade on and what have we learned?. Progress in Neurobiology.

[b33-186-196] Kubota M, Nakazaki S, Hirai S (2001). Alcohol consumption and frontal lobe shrinkage: Study of 1,432 non-alcoholic subjects. Journal of Neurology, Neurosurgery, and Psychiatry.

[b34-186-196] Lewohl JM, Wang L, Miles MF (2000). Gene expression in human alcoholism: Microarray analysis of frontal cortex. Alcoholism: Clinical and Experimental Research.

[b35-186-196] Lisman SA (1974). Alcoholic “blackout”: State dependent learning?. Archives of General Psychiatry.

[b36-186-196] Lister RG, Gorenstein C, Fisher-Flowers D (1991). Dissociation of the acute effects of alcohol on implicit and explicit memory processes. Neuropsychologia.

[b37-186-196] Little HJ (1999). The contribution of electrophysiology to knowledge of the acute and chronic effects of ethanol. Pharmacology and Therapeutics.

[b38-186-196] Martin SJ, Morris RG (2002). New life in an old idea: The synaptic plasticity and memory hypothesis revisited. Hippocampus.

[b39-186-196] Mello NK, Gross MM (1973). Short-term memory function in alcohol addicts during intoxication. Alcohol Intoxication and Withdrawal: Experimental Studies.

[b40-186-196] Mintzer MZ, Griffiths RR (2002). Alcohol and triazolam: Differential effects on memory, psycho-motor performance and subjective ratings of effects. Behavioural Pharmacology.

[b41-186-196] Morrisett RA, Swartzwelder HS (1993). Attenuation of hippocampal long-term potentiation by ethanol: A patch-clamp analysis of glutamatergic and GABAergic mechanisms. Journal of Neuroscience.

[b42-186-196] Moselhy HF, Georgiou G, Kahn A (2001). Frontal lobe changes in alcoholism: A review of the literature. Alcohol and Alcoholism.

[b43-186-196] Mumenthaler MS, Taylor JL, O’Hara R (1999). Gender differences in moderate drinking effects. Alcohol Research & Health.

[b44-186-196] Nelson EC, Madden PAF, Bucholz KK (2004). Genetic epidemiology of alcohol-induced block-outs. Archives of General Psychiatry.

[b45-186-196] Nordby K, Watten RG, Raanaas RK, Magnussen S (1999). Effects of moderate doses of alcohol on immediate recall of numbers: Some implications for information technology. Journal of Studies on Alcohol.

[b46-186-196] O’Malley PM, Johnston LD (2002). Epidemiology of alcohol and other drug use among American college students. Journal of Studies on Alcohol.

[b47-186-196] Orr G, Rao G, Houston FP (2001). Hippocampal synaptic plasticity is modulated by theta rhythm in the fascia dentata of adult and aged freely behaving rats. Hippocampus.

[b48-186-196] Otten LJ, Henson RNA, Rugg MD (2001). Depth of processing effects on neural correlates of memory encoding: Relationship between findings from across- and within-task comparisons. Brain.

[b49-186-196] Parker ES, Birnbaum IM, Noble EP (1976). Alcohol and memory: Storage and state dependency. Journal of Verbal Learning and Verbal Behaviour.

[b50-186-196] Peoples RW, Stewart RR (2000). Alcohols inhibit N-methyl-*d*-aspartate receptors via a site exposed to the extracellular environment. Neuropharmacology.

[b51-186-196] Peoples RW, Li C, Weight FF (1996). Lipid vs. protein theories of alcohol action in the nervous system. Annual Review of Pharmacology and Toxicology.

[b52-186-196] Poikolainen K (1982). Blackouts increase with age, social class and the frequency of intoxication. Acta Neurological Scandinavica.

[b53-186-196] Pyapali GK, Turner DA, Wilson WA, Swartzwelder HS (1999). Age and dose-dependent effects of ethanol on the induction of hippocampal long-term potentiation. Alcohol.

[b54-186-196] Ranganath C, Johnson MK, D’Esposito M (2003). Prefrontal activity associated with working memory and episodic long-term memory. Neuropsychologia.

[b55-186-196] Ryback RS (1970). Alcohol amnesia: Observations in seven drinking inpatient alcoholics. Quarterly Journal of Studies on Alcohol.

[b56-186-196] Ryback RS (1971). The continuum and specificity of the effects of alcohol on memory. Quarterly Journal of Studies on Alcohol.

[b57-186-196] Saum CA, Inciardi JA (1997). Rohypnol misuse in the United States. Substance Use & Misuse.

[b58-186-196] Schummers J, Browning MD (2001). Evidence for a role for GABA(A) and NMDA receptors in ethanol inhibition of long-term potentiation. Brain Research.

[b59-186-196] Scoville WB, Milner B (1957). Loss of recent memory after bilateral hippocampal lesions. Journal of Neurology, Neurosurgery, and Psychiatry.

[b60-186-196] Shastri L (2002). Episodic memory and cortico-hippocampal interactions. Trends in Cognitive Sciences.

[b61-186-196] Silvers JM, Tokunaga S, Berry RB (2003). Impairments in spatial learning and memory: Ethanol, allopregnanolone and the hippocampus. Brain Research Reviews.

[b62-186-196] Steffensen SC, Yeckel MF, Miller DR (1993). Ethanol-induced suppression of hippocampal long-term potentiation is blocked by lesions of the septohippocampal nucleus. Alcoholism: Clinical and Experimental Research.

[b63-186-196] Swartzwelder HS, Wilson WA, Tayyeb MI (1995). Differential sensitivity of NMDA receptor-mediated synaptic potentials to ethanol in immature vs. mature hippocampaus. Alcoholism: Clinical and Experimental Research.

[b64-186-196] Tapert SF, Brown GG, Kindermann SS (2001). fMRI measurement of brain dysfunction in alcohol-dependent young women. Alcoholism: Clinical and Experimental Research.

[b65-186-196] Tiihonen J, Kuikka J, Hakola P (1994). Acute ethanol-induced changes in cerebral blood flow. American Journal of Psychiatry.

[b66-186-196] Volkow ND, Mullani N, Gould L (1988). Effects of acute alcohol intoxication on cerebral blood flow measured with PET. Psychiatry Research.

[b67-186-196] Wall PM, Messier C (2001). The hippocampal formation–orbitomedial prefrontal cortex circuit in the attentional control of active memory. Behavioral Brain Research.

[b68-186-196] Wang GJ, Volkow ND, Franceschi D (2000). Regional brain metabolism during alcohol intoxication. Alcoholism: Clinical and Experimental Research.

[b69-186-196] Wechsler H, Lee JE, Kuo M (2002). Trends in college binge drinking during a period of increased prevention efforts. Findings from 4 Harvard School of Public Health College Alcohol Study surveys: 1993–2001. Journal of American College Health.

[b70-186-196] Weissenborn R, Duka T (2000). State-dependent effects of alcohol on explicit memory: The role of semantic associations. Psychopharmacology.

[b71-186-196] Weissenborn R, Duka T (2003). Acute alcohol effects on cognitive function in social drinkers: Their relationship to drinking habits. Psychopharmacology.

[b72-186-196] Westrick ER, Shapiro AP, Nathan PE, Brick J (1988). Dietary tryptophan reverses alcohol-induced impairment of facial recognition but not verbal recall. Alcoholism: Clinical and Experimental Research.

[b73-186-196] White AM, Best PJ (2000). Effects of ethanol on hippocampal place-cell and interneuron activity. Brain Research.

[b74-186-196] White AM, Simson PE, Best PJ (1997). Comparison between the effects of ethanol and diazepam on spatial working memory in the rat. Psychopharmacology.

[b75-186-196] White AM, Ghia AJ, Levin ED, Swartzwelder HS (2000a). Binge-pattern ethanol exposure in adolescent and adult rats: Differential effects on subsequent ethanol exposure. Alcoholism: Clinical and Experimental Research.

[b76-186-196] White AM, Matthews DB, Best PJ (2000b). Ethanol, memory and hippocampal function: A review of recent findings. Hippocampus.

[b77-186-196] White AM, Jamieson-Drake DW, Swartzwelder HS (2002a). Prevalence and correlates of alcohol-induced blackouts among college students: Results of an e-mail survey. Journal of American College Health.

[b78-186-196] White AM, Roberts DC, Best PJ (2002b). Context-specific tolerance to the ataxic effects of ethanol. Pharmacology Biochemistry and Behavior.

[b79-186-196] White AM, Signer ML, Kraus CL, Swartzwelder HS (2004). Experiential aspects of alcohol-induced blackouts among college students. American Journal of Drug and Alcohol Abuse.

[b80-186-196] Zola-Morgan S, Squire LR, Amaral DG (1986). Human amnesia and the medial temporal lobe region: Enduring memory impairment following a bilateral lesion limited to field CA1 of the hippocampus. Journal of Neuroscience.

